# Supplementary material for: Association between oral microbiome and seven types of cancers in East Asian population: a two-sample Mendelian randomization analysis
Source: Front Mol Biosci. 2023 Nov 21;10:1327893. doi: 10.3389/fmolb.2023.1327893 (PMC10702768; doi:10.3389/fmolb.2023.1327893)
Supplement: Supplementary file 1 [file DataSheet1.ZIP › Supplementary Materials/MR plots of saliva/saliva═╝/colorectal cancer/pheno.3289_to_colorectal cancer_leave_one_out.pdf]

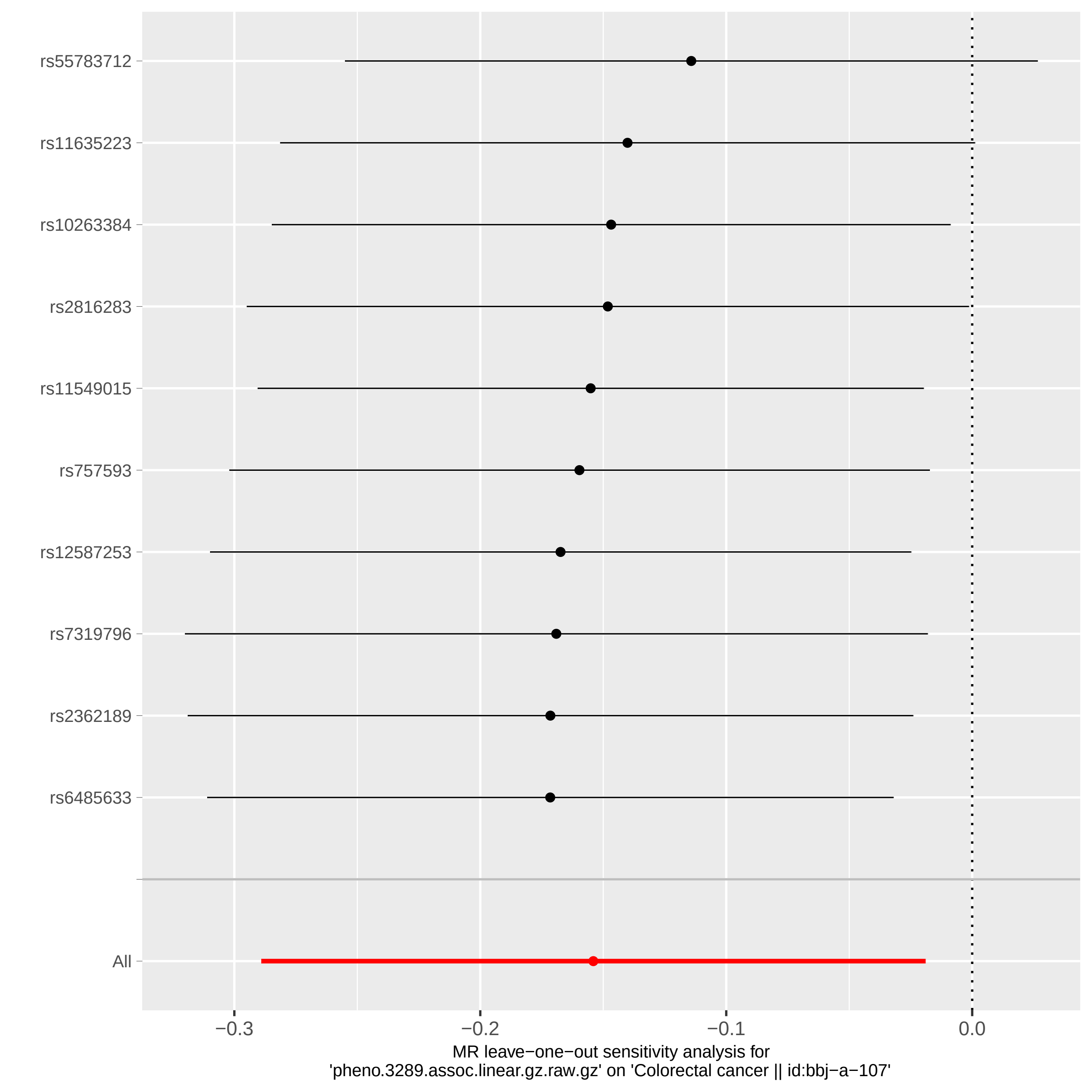
rs55783712
rs11635223
rs10263384
rs2816283
rs11549015
rs757593
rs12587253
rs7319796
rs2362189
rs6485633
All
−0.3
−0.2
−0.1
0.0
MR leave−one−out sensitivity analysis for
'pheno.3289.assoc.linear.gz.raw.gz' on 'Colorectal cancer || id:bbj−a−107'
